# Detection of CWD prions in naturally infected white-tailed deer fetuses and gestational tissues by PMCA

**DOI:** 10.1038/s41598-021-97737-y

**Published:** 2021-09-15

**Authors:** Francisca Bravo-Risi, Paulina Soto, Thomas Eckland, Robert Dittmar, Santiago Ramírez, Celso S. G. Catumbela, Claudio Soto, Mitch Lockwood, Tracy Nichols, Rodrigo Morales

**Affiliations:** 1 Department of Neurology, The University of Texas Health Science Center at Houston, 6431 Fannin St. MSB 7.128, Houston, TX 77030 USA; 2 Texas Parks and Wildlife Department, Kerrville, TX USA; 3 United States Department of Agriculture, Animal Plant Health Inspection Service, Veterinary Services, Fort Collins, CO 80526 USA; 4 Centro Integrativo de Biologia y Quimica Aplicada (CIBQA), Universidad Bernardo O’Higgins, Santiago, Chile

**Keywords:** Biochemistry, Biotechnology, Environmental sciences, Biomarkers

## Abstract

Chronic wasting disease (CWD) is a prevalent prion disease affecting cervids. CWD is thought to be transmitted through direct animal contact or by indirect exposure to contaminated environmental fomites. Other mechanisms of propagation such as vertical and maternal transmissions have also been suggested using naturally and experimentally infected animals. Here, we describe the detection of CWD prions in naturally-infected, farmed white-tailed deer (WTD) fetal tissues using the Protein Misfolding Cyclic Amplification (PMCA) technique. Prion seeding activity was identified in a variety of gestational and fetal tissues. Future studies should demonstrate if prions present in fetuses are at sufficient quantities to cause CWD after birth. This data confirms previous findings in other animal species and furthers vertical transmission as a relevant mechanism of CWD dissemination.

## Introduction

Chronic wasting disease (CWD) is a prion disease affecting several cervid species^1–3^. CWD clinical signs (including lack of fear of people, polydipsia, polyurea, weight loss, progressive wasting/weakness, and others) are due to the accumulation of neurotoxic misfolded forms of the prion protein (PrP^Sc^) in specific areas of the brain^1,3^. PrP^Sc^ aggregates are also present in several peripheral tissues, including lymphoid tissues, peripheral nerves, muscle, blood and skin, among others^4–7^. The presence of prion infectivity in peripheral tissues is considerably lower compared to the brain^4,8^. Moreover, PrP^Sc^ aggregates are the infectious particles causing CWD^9^.

CWD can reach high incidence in certain populations. Extensive evidence demonstrates that naïve animals are infected through direct contact with diseased animals or by indirect exposure to contaminated fomites^9^. CWD prions are known to exist in low quantities in excreta^10–12^. However, the continuous release of infectious prion particles during most of the CWD prion incubation period and clinical disease make them relevant for disease transmission^13,14^. The latter is strongly supported by the fact that prions have high affinity for certain natural and manmade materials (e.g., soil, plants, stainless steel, polypropylene, among others)^15–17^, and are resistant to degradation^18,19^. Nevertheless, other possible routes of transmission, such as sexual and vertical routes, still present questions.

The potential for sexual and maternal routes in prion disease transmission has been studied in different systems, including naturally or experimentally infected sheep^20–22^, goats^23,24^, cattle^25,26^, and rodent models^27^. Many experiments in sheep, goats and Syrian hamsters suggest that sexual contact is not an efficient mean to transmit prion disease^27,28^. However, PrP^Sc^ has been identified in several reproductive and gestation-related tissues such as testes, ovaries, uterus, placenta and amniotic fluid^21,29,30^. In addition, progeny from infected deer, sheep and cattle are known to have increased risks to develop prion disease^26,31,32^. Abnormal accumulation of prion protein, seeding activity and/or prion infectivity have been identified in fetal tissues from sheep^33^ and elk^30^. Mother-to-offspring prion transmission appears to be prion-strain specific as evidence in other animal species including humans, Syrian hamsters and sheep infected with the classical bovine spongiform encephalopathy (BSE) agent show that progeny from infected females at the moment of gestation do not develop prion disease in the long-term^22,27^. For the specific case of CWD, previous reports demonstrated the presence of PrP^Sc^ in gestational tissues of naturally infected elk^30^ and experimentally-infected muntjac deer^29^. In both cases, prion identification using either the Protein Misfolding Cyclic Amplification (PMCA) technique, or immunohistochemistry (IHC) provided convincing evidence of prion accumulation in fetal tissues. Nevertheless, whether this occurs in naturally infected white-tailed deer (WTD) is still unknown.

Here, we report the presence of seeding competent CWD prions in fetal tissues collected from naturally prion-infected farmed WTD does using PMCA. The results presented in this article confirm the presence of CWD prions in fetal tissues from naturally infected farmed WTD dams suggesting that CWD could be transferred from mother to offspring.

## Materials and methods

### Sample collection and preparation

Whole gestational sacs were tied off and removed intact *postmortem* from the does, frozen-shipped to UTHealth facilities and stored at − 80 °C. Whole gestational sacs were slowly thawed by placing them at 4 °C until tissues were ready for collection. Maternal and fetal tissues were dissected and collected. Tissues and fluids collected included the following: (1) fetal: brain, lung, kidney, liver, popliteal lymph node, thymus, submandibular lymph node, spleen, parotid, testis, uterus, peripheral nerves; (2) gestational: amniotic fluid, umbilical cord, amniotic sac, placenta, and cotyledons (maternal and fetal side, one per doe/fetus). Clean (prion-free/new) instruments were used to dissect and collect each tissue. Amniotic fluid was collected using disposable syringes. All tissue samples were homogenized at 10% w/v in PBS containing a cocktail of protease inhibitors (Roche, Basel, Switzerland). Fetal samples were not tested by IHC as they were frozen and not properly stored for this procedure.

### Western blotting and proteinase K (PK) treatments on fetal and gestational tissues

To visualize the content of the normal prion protein (PrP^C^), proteins present in the tissues and fluids mentioned above were fractioned in NuPAGE 12%, Bis–Tris gels (Invitrogen, Carlsbad, CA, USA) and transferred to nitrocellulose membranes (GE Healthcare Amersham, Chicago, IL, USA). Membranes were blocked using 5% w/v non-fat milk solution and probed with monoclonal 6H4 antibody (Prionics, Zurich, Switzerland) at 1:12,500 dilution. After washing, membranes were incubated with secondary antibody polyclonal Anti-Mouse IgG (whole molecule)–Peroxidase antibody produced in sheep (Sigma-Aldrich, Saint Louis, MO, USA) at 1:3,000 dilution. Membranes were washed and developed using ECL (GE Healthcare Amersham, Chicago, IL, USA) following manufacturer’s recommendations. Additionally, the same tissue homogenates were treated with PK (Sigma-Aldrich, Saint Louis, MO, USA) at a 100 µg/mL final concentration to assess for potential presence of disease-associated prion proteins (PrP^Sc^). Conditions used for PK digestion were 37 °C with shaking for 90 min. PK reactions were stopped by the addition of LDS sample buffer and heating (95 °C for 10 min).

### PMCA

The PMCA procedure was performed as extensively described in our previous publications^34^ and specifically adapted for CWD^6,35^. Briefly, brains from Tg1536 mice were homogenized at 10% w/v in PMCA conversion buffer [1× PBS, 150 mM NaCl, 1% Triton X-100, and protease inhibitors cocktail (Roche, Basel, Switzerland)] to generate the PMCA substrate. Aliquots (90 µL) of this PMCA substrate were mixed with either 10 µL of WTD tissue homogenates or amniotic fluids and submitted to 144 cycles of incubation and sonication (each PMCA cycle consisting of 29 min and 40 s of incubation, and 20 s of sonication). Resulting samples were subjected to two additional rounds of PMCA (96 cycles each) by mixing 10 µL of the PMCA products of each round with new PMCA substrate (90 µL). PMCA products were treated with PK as described above and examined by western blotting. As controls, each PMCA reaction set (testing approximately 50 samples) included serial dilutions of a CWD brain of known PMCA activity and at least four unseeded reactions. Each sample was tested in duplicate by two different investigators and the results were evaluated at the third round of PMCA. Considering that all unseeded PMCA reactions in our study resulted in negative PrP^Sc^ signals, CWD presence was assumed if at least one of the replicates resulted in positive detection.

### Ethics approval

WTD (gestational and fetal) tissues were collected *postmortem* by Texas Parks and Wildlife Department (TPWD) and United States Department of Agriculture Animal Plant Health Inspection Service (USDA/APHIS) personnel as part of depopulation procedures for a CWD positive farm. Fetuses (n = 5, 1 female and 4 males) were collected from two asymptomatic WTD does. Gestation dates were estimated between 128–130 (twins) and 154–180 (triplets) days of gestation according to forehead-rump length measurements^36^ and hair pigmentation patterns. Additional information on the pregnant females and fetuses collected is provided in Table 1. Brains from male and female homozygous Tg1536 mice, expressing the WTD version of the prion protein^37^, were used as PMCA substrate (explained above). Mice were bred in approved facilities and euthanized by CO_2_ inhalation following federal and local regulations, and approved by the Animal Welfare Committee of The University of Texas Health Science Center at Houston (UTHealth, protocol AWC-20-0065). Animal manipulations described in this article follows the recommendations stated in the ARRIVE guidelines (https://arriveguidelines.org/). The experiments listed in this manuscript did not involve animal experimentations. UTHealth researchers have USDA approval to receive and work with white-tailed deer-derived samples.

**Table 1:** White-tailed deer fetuses (twins and triplets) collected from two prion infected does.

Fetus ID	Gestation time (days)^a^	Sex	PrP polymorphisms at position 96	Fetal tissues collected
Twin001	128–130	Female	GG	Brain; parotid; peripheral nerves; kidney; spleen; liver; uterus; lymph nodes (submandibular and subscapular)
Twin002		Male	GS	Brain; parotid; peripheral nerves; kidney; spleen; liver; lung; testis; subscapular lymph node
Triplet003	154–180	Male	GS	Brain; parotid; peripheral nerves; kidney; spleen; liver; lung; testis; thymus; popliteal lymph node
Triplet004		Male	GG	Brain; parotid; peripheral nerves; kidney; spleen; liver; lung; testis; lymph nodes (submandibular and popliteal)
Triplet005		Male	GG	Brain; parotid; kidney; spleen; liver; testis; thymus; submandibular lymph node

^a^Estimated gestation time by forehead-rump length measurements and hair pigmentation patterns.

## Results

Tissues from five fetuses (Table 1), and associated gestational structures, were collected from two naturally CWD-infected farmed WTD does carrying twins or triplets. The does were both asymptomatic and their CWD status was confirmed by IHC in retropharyngeal lymph nodes and obex as recommended by the USDA (https://www.aphis.usda.gov/aphis/ourfocus/animalhealth/nvap/NVAP-Reference-Guide/Control-and-Eradication/Chronic-Wasting-Disease). These two WTD females were considered to be at the late stages of their CWD prion incubation periods as positive signals were found in both tissues^38^.

**Figure 1:**
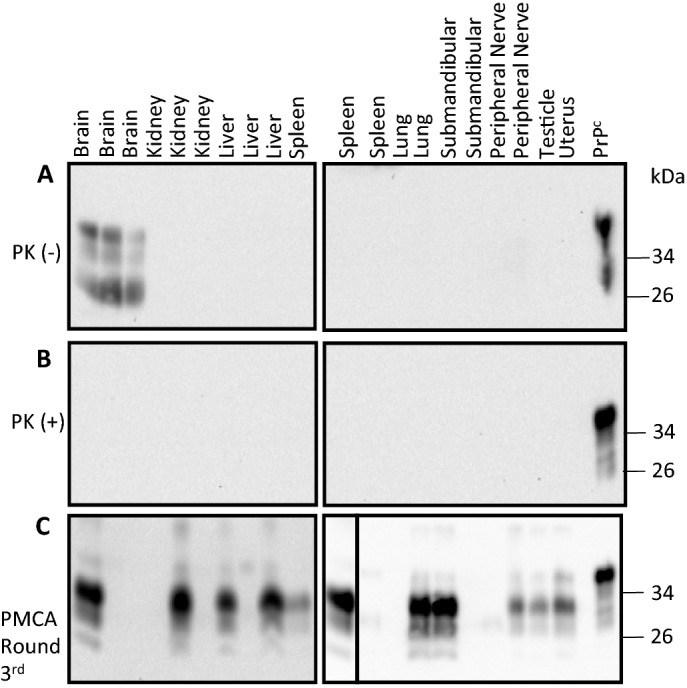
PrP^C^ and PrP^Sc^ screening in white-tailed deer fetal tissues using western blot and PMCA. Western blot analysis of representative fetal tissue samples prior (**A**) and after (**B**) PK treatment. (**C**) Results from the same representative samples depicted in (**A**) and (**B**) after PMCA analysis. Numbers at the right of each panel represent molecular weight markers (in KDa). “PrP^C^” denotes brain extracts from Tg1536 mice not treated with PK and used as additional molecular weight markers. The solid line within panel 1C denote images taken from different membranes.

Tissues collected from deer fetuses (Table 1) were first analyzed for expression of the physiological/cellular prion protein (PrP^C^) by western blotting. This analysis revealed PrP^C^ expression only in fetal brains (Fig. 1A). PK treatment on these samples did not provide signals associated with PrP^Sc^ in any of the tissues studied, suggesting that these specimens are devoid of large CWD infectivity titers (Fig. 1B). The same tissues were later tested for the presence of seeding competent PrP^Sc^ using PMCA (Fig. 1C). In the larger fetal organs, PMCA seeding activity was identified in livers (4/5 fetuses), lungs (2/3 fetuses) and kidneys (2/5 fetuses). Brains presented the lowest degree of seeding activity as only 1 out of 5 fetal brains displayed positive PMCA signals. Seeding activity was also detected in most of the lymphoid tissues analyzed in this study (spleens, thymus, mesenteric-, submandibular- and subscapular-lymph nodes). Popliteal lymph nodes were the only lymphoid tissues negative for PrP^Sc^ detection, although only samples from 2 out of 5 fetuses were collected and analyzed in this study. Surprisingly, a high proportion of fetal reproductive tissues (testes and uterus) were CWD positive after the PMCA screening. Analysis of peripheral nerves showed that only 2/4 samples analyzed contained seeding competent CWD prions. All these results are summarized in Table 2. Importantly, we did not observe any marked difference between the twin and triplet groups in terms of PrP^Sc^ distribution, although we acknowledge that number of animals in this study is low.

**Table 2:** Summary of PMCA screening in fetal and gestational tissues and fluids collected from two pregnant white-tailed deer does.

	Tissue type	Positive PMCA detection/fetal tissues and fluids analyzed
Fetal organs	Brain	1/5
	Lungs	2/3
	Kidney	2/5
	Liver	4/5
	Popliteal lymph node	0/2
	Thymus	1/2
	Submandibular lymph node	2/3
	Spleen	4/5
	Subscapular lymph node	2/2
	Parotid	1/5
	Testis	3/4
	Uterus	1/1
	Peripheral nerves	2/4
Gestational tissues/fluids	Amniotic fluid	0/5
	Umbilical cord	2/5
	Amniotic sac	3/4
	Placenta	1/1
	Cotyledon (fetal side)	2/2
	Cotyledon (maternal side)	2/2

**Figure 2:**
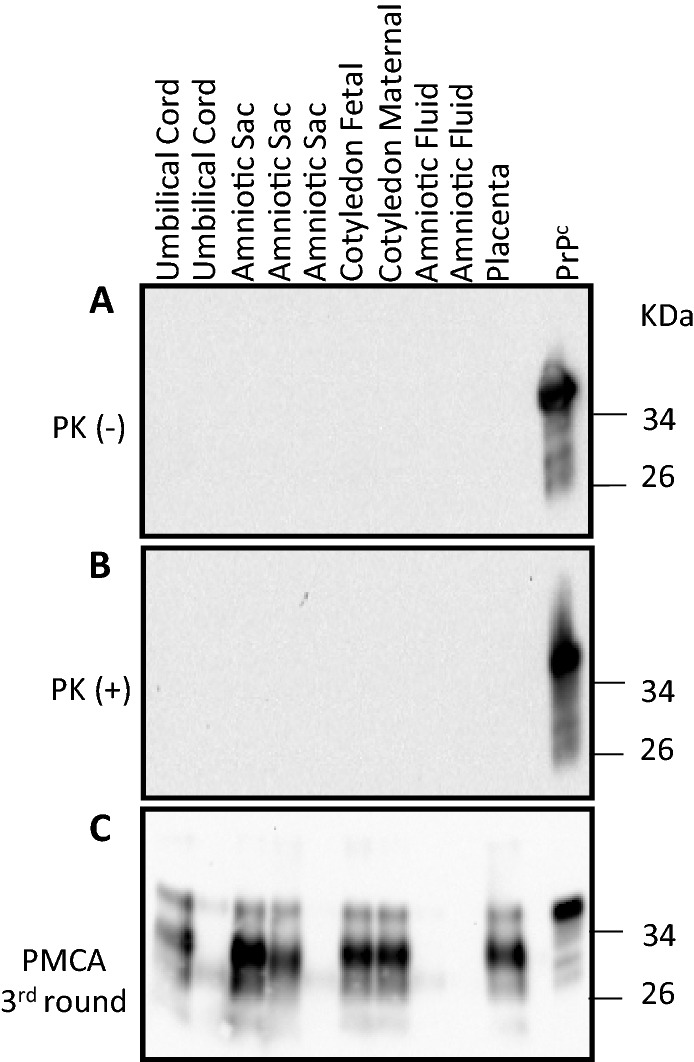
Identification of PrP^Sc^ in white-tailed deer gestational tissues and fluids by western blot and PMCA. Western blot analysis of representative gestational tissue samples prior (**A**) and after (**B**) PK treatment. (**C**) PMCA results from the same representative samples depicted in (**A**) and (**B**) after three PMCA rounds. Numbers at the right of each panel represent molecular weight markers (in KDa). “PrP^C^” denotes brain extracts from Tg1536 mice not treated with PK and used as additional molecular weight markers.

We also tested gestational tissues and fluids of the does (umbilical cords, amniotic sacs, placentas, cotyledons and amniotic fluids) for their presence of CWD prions using western blots and PMCA. Homogenates from these samples, as well as amniotic fluids, did not display PrP^C^ as assessed by western blots (Fig. 2A). Similar to all fetal tissues, no PK-resistant PrP signals were detected in any of the gestational tissues analyzed in this study (Fig. 2B). Further analyses by PMCA revealed presence of CWD prions in umbilical cords, amniotic sacs, cotyledons (fetal and maternal sides) and placentas, in agreement with previously published data^29,30^. In this screening, all amniotic fluid samples analyzed provided negative results in PMCA (Fig. 2). Original data files associated with this study are displayed in Supplementary Information 1.

Some of the fetal and maternal tissues tested in this study were genotyped to identify possible polymorphic variations at position 96 of the prion protein (Table 1). It has been largely described that variability at this level alters CWD susceptibility^39^ and modulate the pathogenicity of infectious prions^40^. Both does were homozygous for the most common polymorphic variation (glycine, G). While three fetuses mirrored the does in this aspect, the remaining two carried one copy of the least common PrP isoform coding a serine (S) at this position (Table 1). We did not observe any obvious effect of PrP polymorphic variation in terms of prion detection, although we acknowledge that numbers were low to draw definitive conclusions.

As mentioned above, PMCA results were evaluated at the third PMCA round. However, most of the tissues considered PMCA-positive provided the same results in the first PMCA round. The only exceptions were found for one specimen of the following sample types: umbilical cord, spleen, submandibular lymph node and subscapular lymph node (data not shown). Importantly, tissues displaying this behavior were scattered across different subjects and similar behaviors were not observed in equivalent specimens from other fetuses.

## Discussion

CWD is rapidly expanding in both captive and wild cervid populations. While direct animal contact and environmental contamination provide reasonable explanations on how this disease is transmitted, evidence involving fetal infection and maternal exposure suggest that these routes may be relevant for disease transmission. Offspring from scrapie-infected sheep has been described as being at increased risk of developing prion disease^32^. Similar outcomes have been described for farmed elk^41^ and experimentally infected muntjac deer^31^. Relevant evidence supporting maternal-offspring CWD transmission include prion seeding activity identified in placenta and gestational fluids from pregnant elk and muntjac deer^29,30^. Importantly, prion detection has been identified in fetal tissues from elk^30^. Controlled experimental conditions in muntjac deer demonstrate that mother-to-offspring transmission is possible for CWD^31^. Our results show that fetal tissues collected from naturally infected CWD-positive asymptomatic farmed WTD females contain seeding competent prions. This suggests that mother-to-offspring prion transmission is a common feature of CWD across different cervid species.

In this report, we communicate the screening of 19 fetal and gestational tissues and fluids for the detection of CWD prions. Relevant CWD positive fetal tissues include liver, kidney, and lymphoid and reproductive tissues. The case of liver and kidney is interesting, as prion accumulation in these tissues is not observed by IHC in adult CWD-symptomatic animals^5^. The presence of CWD prions in fetuses’ sexual tissues is also interesting, especially considering our previous report showing that prion seeding activity is present in the testes of CWD-infected WTD bucks only at the late pre-symptomatic stages^35^. On the contrary, the identification of CWD prions in a large proportion of lymphoid tissues is in alignment with the expected pathophysiology of prions observed in adult animals^2^. This finding suggests that the tropism of infectious prions in lymphoid organs occurs even at fetal stages. However, the results presented in this article do not allow us to conclude whether CWD prions present in fetal tissues came from the mothers through circulation or were generated de novo in the fetuses. The poor detection of CWD prions in fetal brains strongly supports the idea that neuroinvasion (ergo, prion replication) does not occur at fetal stages.

PMCA can detect prions at sub-infectious levels^34,42–44^ and CWD prion amplification by PMCA is able to catch sub-infectious PrP^Sc^ quantities in the first round^6,35^. Whether CWD prions present in fetal tissues exist in quantities large enough to induce clinical CWD after birth cannot be concluded from our results. Previous results in goats show that embryo transfer from infected to naïve females failed to transmit prion disease to offspring^28^, suggesting that if prions in sheep and goat embryos contain prions, they are present in sub-infectious quantities. Nevertheless, it is important to acknowledge that embryos described in those studies were exposed to a prion-infected environment for a restricted time, and either prion absorption and replication by embryos may be limited. The latter assumption is supported by the fact that recipient females were not infected^28^. Nonetheless, similar studies in sheep demonstrated that in utero prion transmission is possible^45^. The presence of prion infectivity in mammary glands, colostrum and milk of sheep suggest that transmission can also occur after birth^46–49^. Future studies detecting prions in mammary glands and milk of deer does will help us to evaluate the different possible scenarios in which CWD can be transmitted from mother to offspring (i.e., in utero vs. milking/nursing). Research in this area is relevant considering that wild WTD CWD-positive does seems more likely to be parents compared to their CWD-negative counterparts^50^.

The results presented in this study show that CWD prions exist in WTD fetuses from naturally infected does. Whether prions in fetal tissues are enough to sustain infectivity after birth, as well as descriptions of the mechanisms governing mother-to-offspring CWD transmission in cervids, should be clarified in future studies. These studies should include the screening of larger number of samples collected from wild and farmed animals affected by different strains of CWD prions, bioassays in susceptible mice to measure infectivity titers, and controlled experiments using pregnant/CWD-infected WTD females.

## Supplementary Information

Supplementary Information 1.
