# Supplementary material for: Detection of CWD prions in naturally infected white-tailed deer fetuses and gestational tissues by PMCA
Source: Sci Rep. 2021 Sep 15;11:18385. doi: 10.1038/s41598-021-97737-y (PMC8443553; doi:10.1038/s41598-021-97737-y)
Supplement: Supplementary file 1 — Supplementary Information 1. [file 41598_2021_97737_MOESM1_ESM.pptx]

## Slide 1
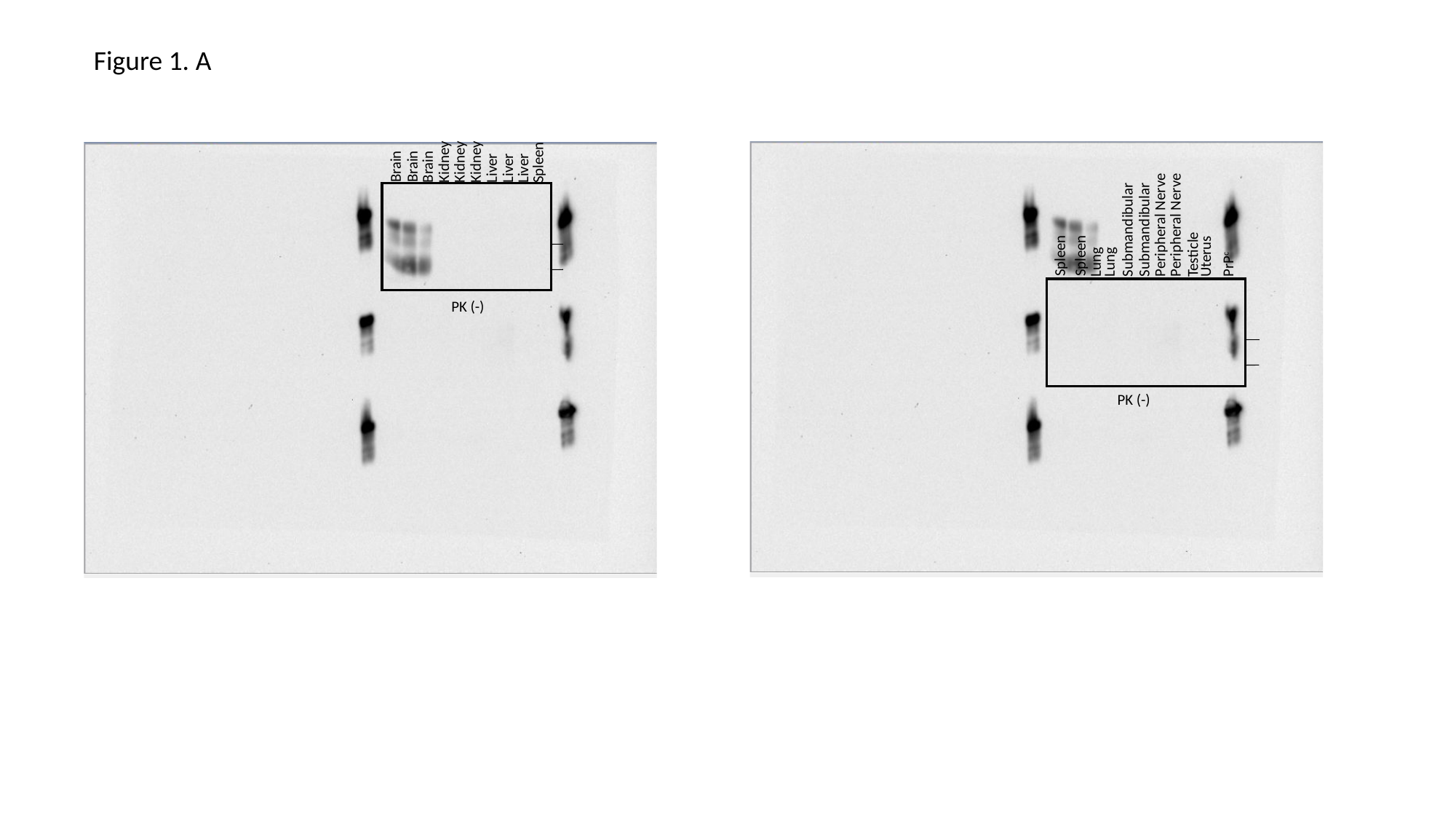

Figure 1. A
Brain
Brain
Brain
Kidney
Kidney
Kidney
Liver
Liver
Liver
Spleen
Peripheral Nerve
Peripheral Nerve
Submandibular
Submandibular
Spleen
Spleen
Lung
PrPc
Lung
Testicle
Uterus
PK (-)
PK (-)

## Slide 2
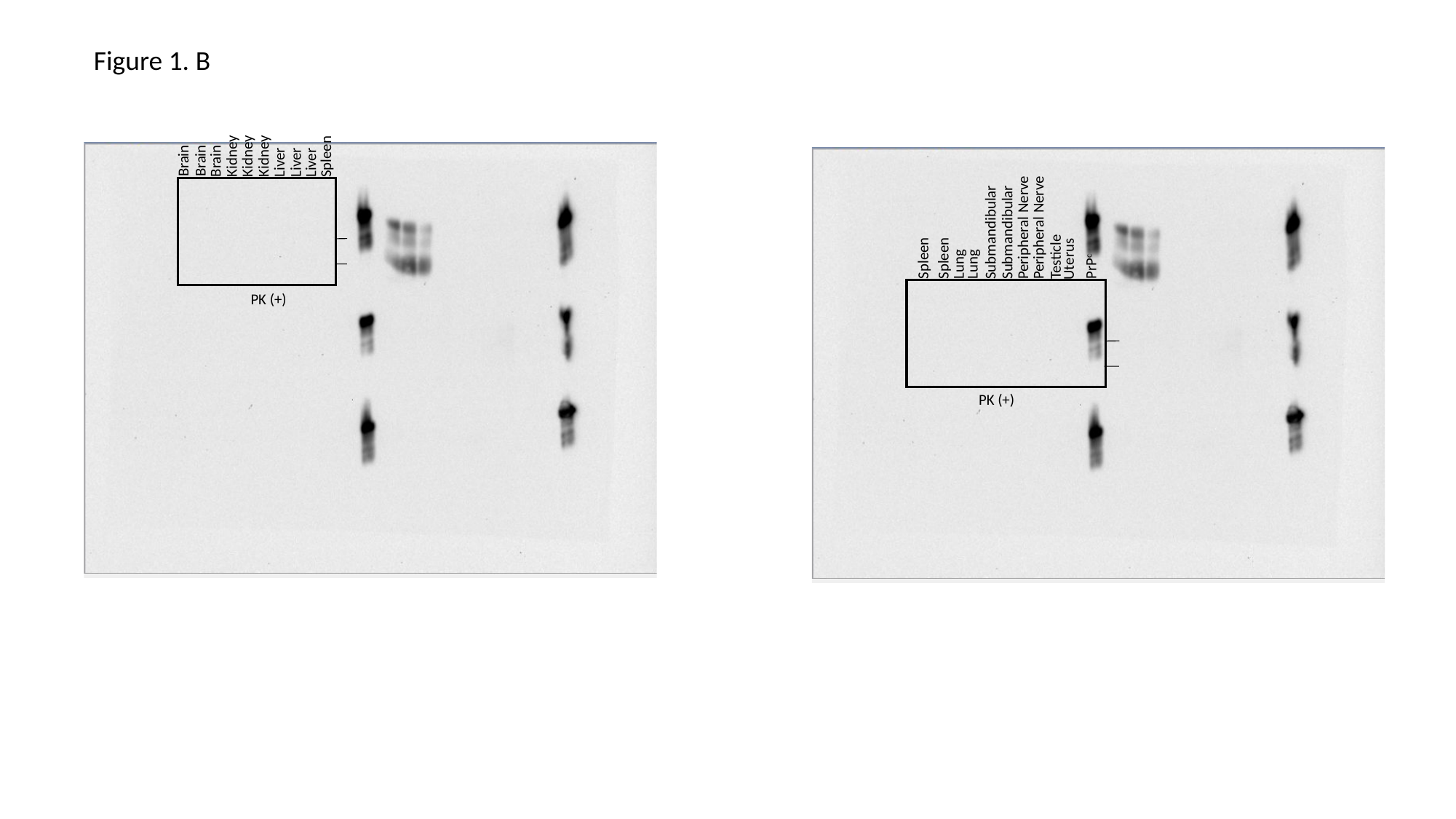

Figure 1. B
Brain
Brain
Brain
Kidney
Kidney
Kidney
Liver
Liver
Liver
Spleen
Peripheral Nerve
Peripheral Nerve
Submandibular
Submandibular
Spleen
Spleen
Lung
PrPc
Lung
Testicle
Uterus
PK (+)
PK (+)

## Slide 3
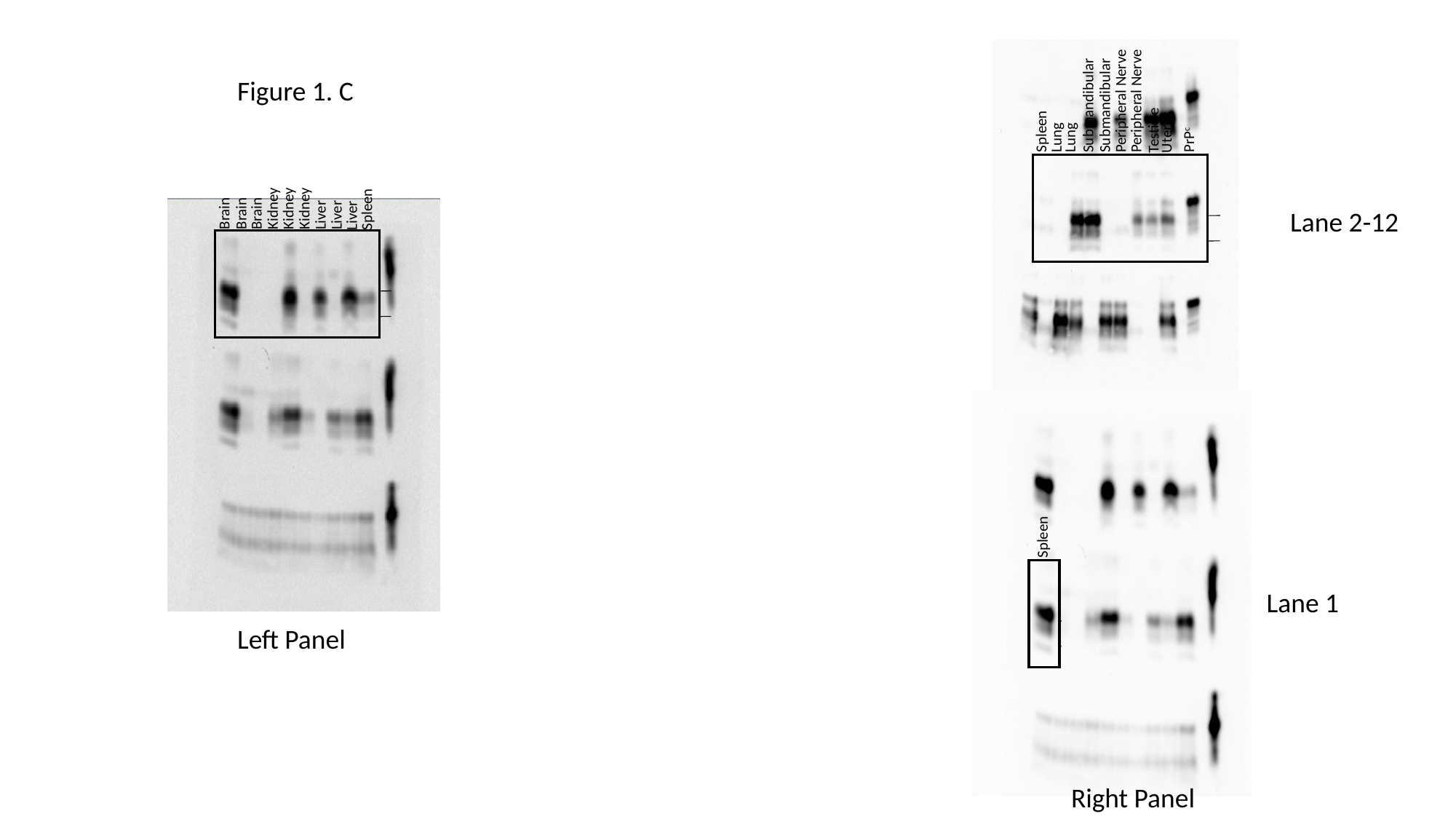

Peripheral Nerve
Figure 1. C
Peripheral Nerve
Submandibular
Submandibular
Spleen
Lung
PrPc
Lung
Testicle
Uterus
Brain
Brain
Brain
Kidney
Kidney
Kidney
Liver
Liver
Liver
Spleen
Lane 2-12
Spleen
Lane 1
Left Panel
Right Panel

## Slide 4
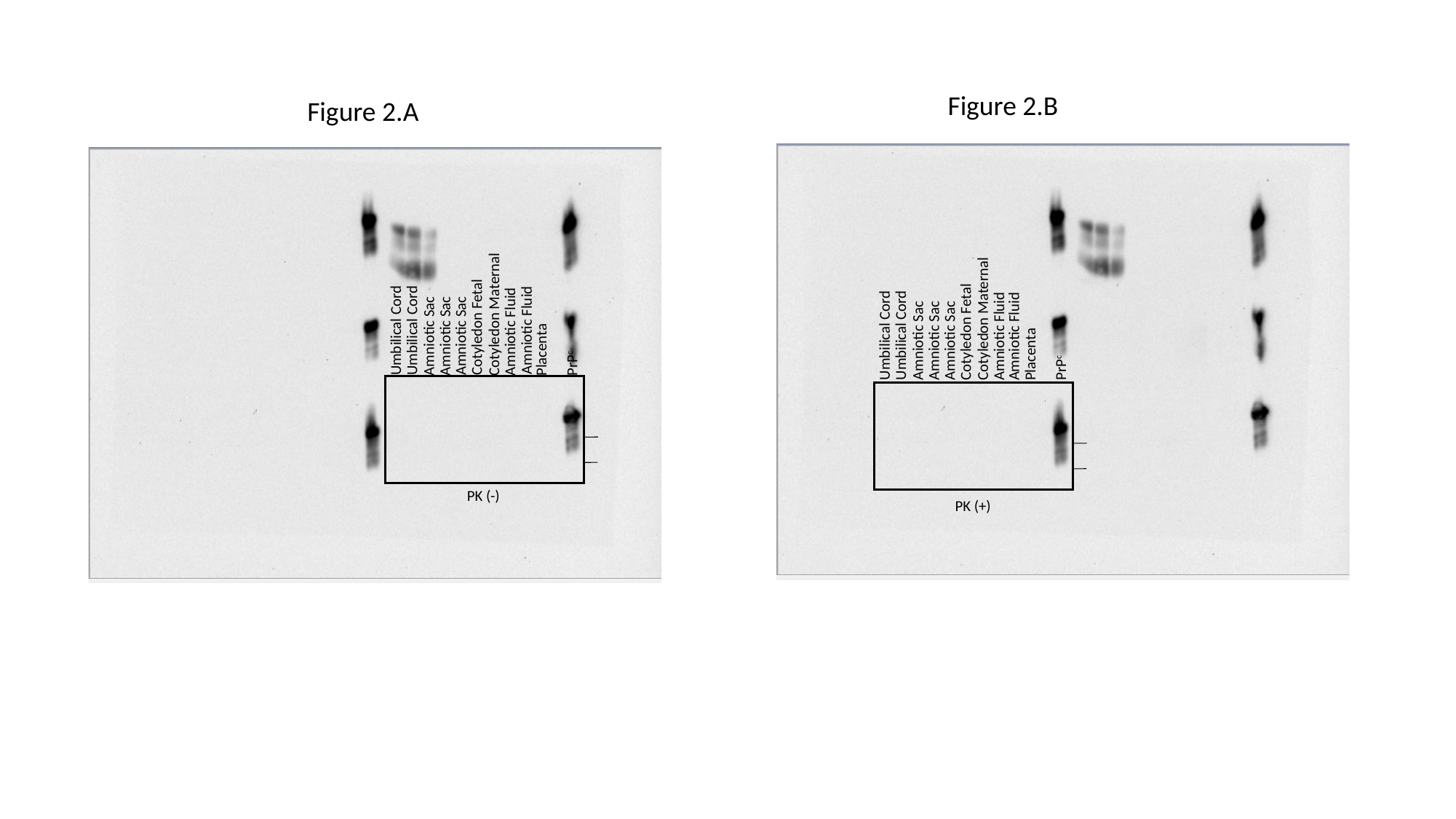

Figure 2.B
Figure 2.A
Cotyledon Maternal
Cotyledon Maternal
Amniotic Fluid
Amniotic Fluid
Amniotic Fluid
Amniotic Fluid
Umbilical Cord
Amniotic Sac
Placenta
Cotyledon Fetal
Umbilical Cord
Amniotic Sac
Amniotic Sac
Umbilical Cord
Placenta
Cotyledon Fetal
Amniotic Sac
Amniotic Sac
Umbilical Cord
Amniotic Sac
PrPc
PrPc
PK (-)
PK (+)

## Slide 5
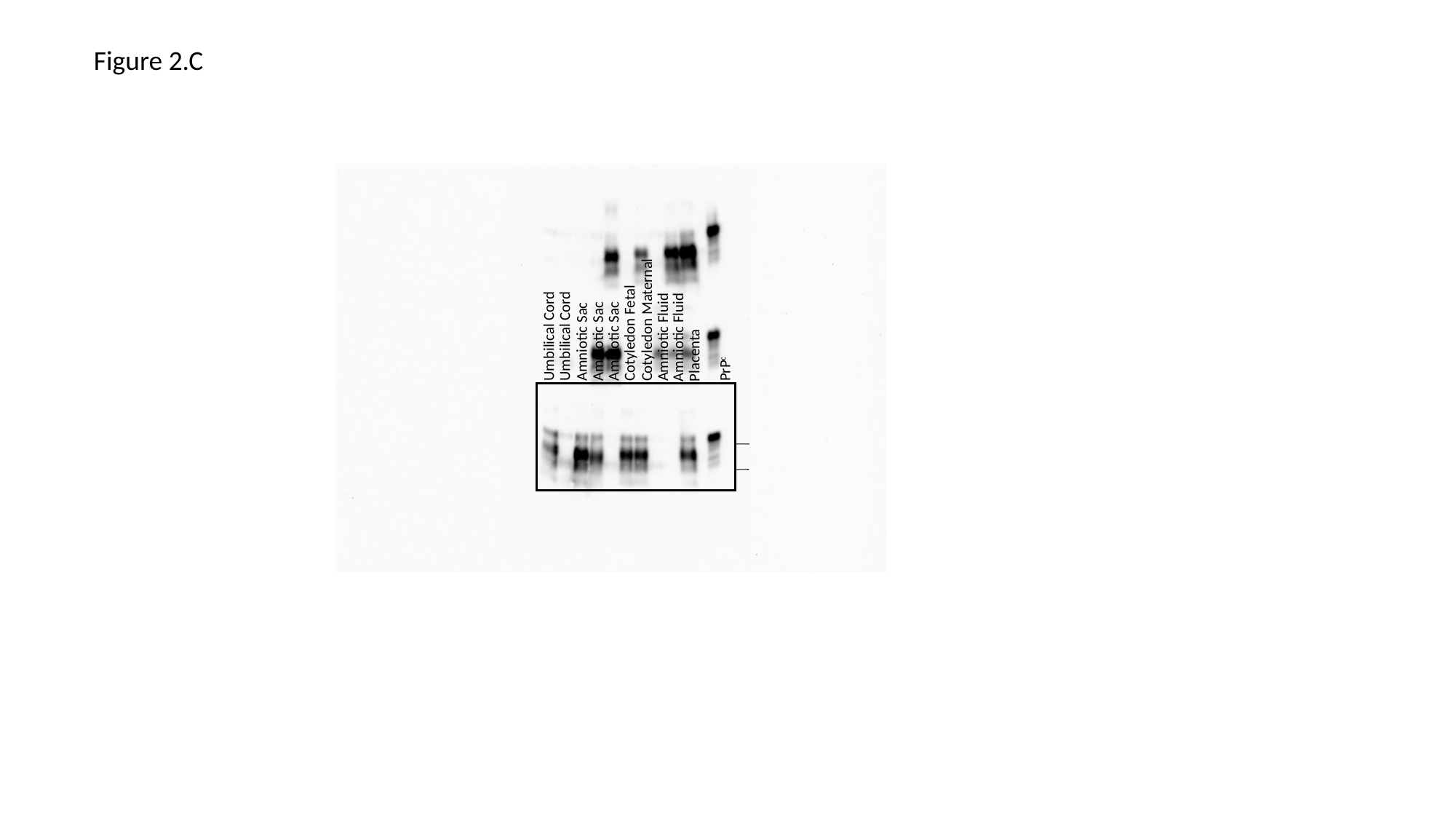

Figure 2.C
Cotyledon Maternal
Amniotic Fluid
Amniotic Fluid
Umbilical Cord
Amniotic Sac
Placenta
Cotyledon Fetal
Amniotic Sac
Umbilical Cord
Amniotic Sac
PrPc

## Slide 6
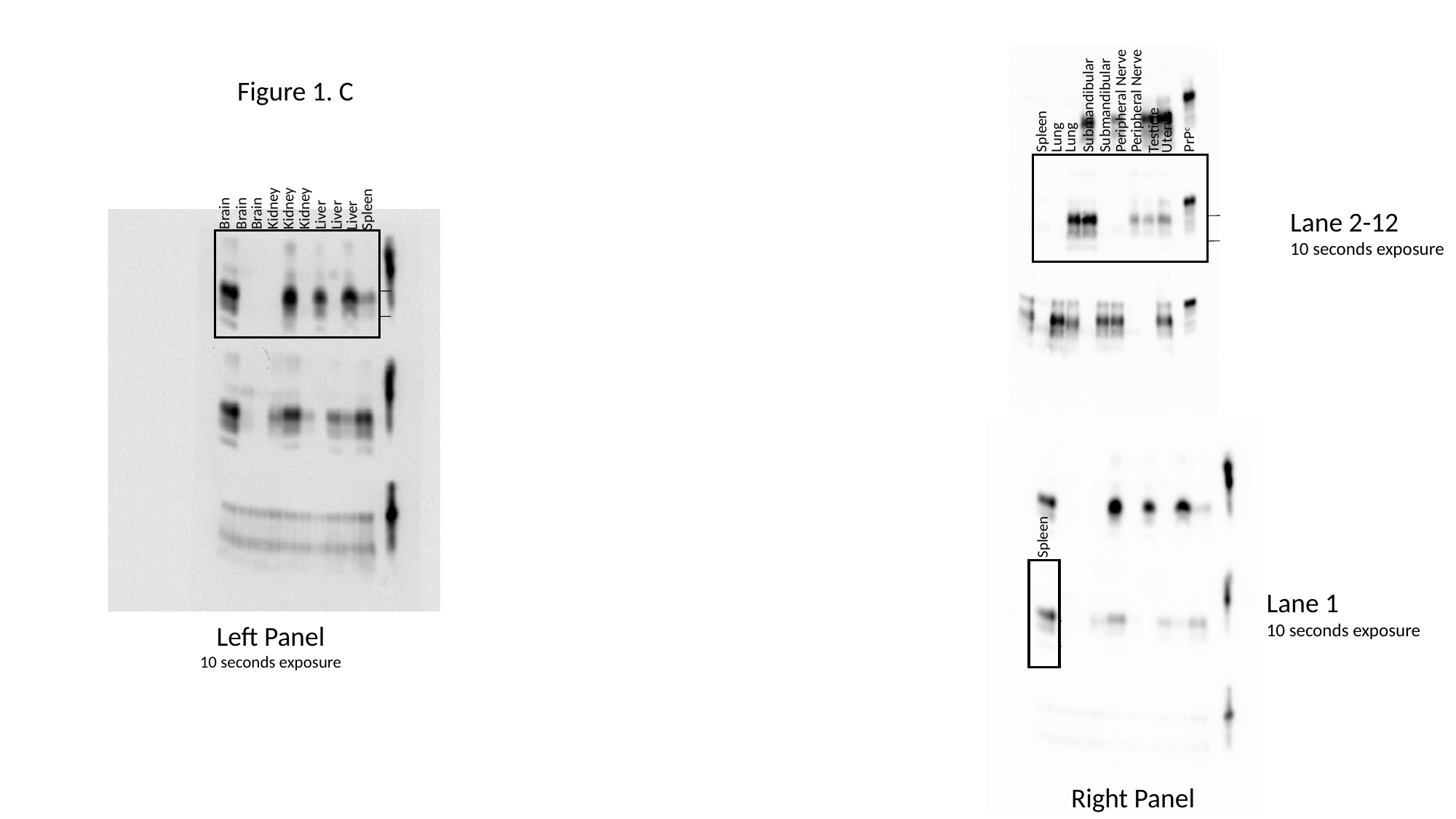

Peripheral Nerve
Figure 1. C
Peripheral Nerve
Submandibular
Submandibular
Spleen
Lung
PrPc
Lung
Testicle
Uterus
Brain
Brain
Brain
Kidney
Kidney
Kidney
Liver
Liver
Liver
Spleen
Lane 2-12
10 seconds exposure
Spleen
Lane 1
10 seconds exposure
Left Panel
10 seconds exposure
Right Panel

## Slide 7
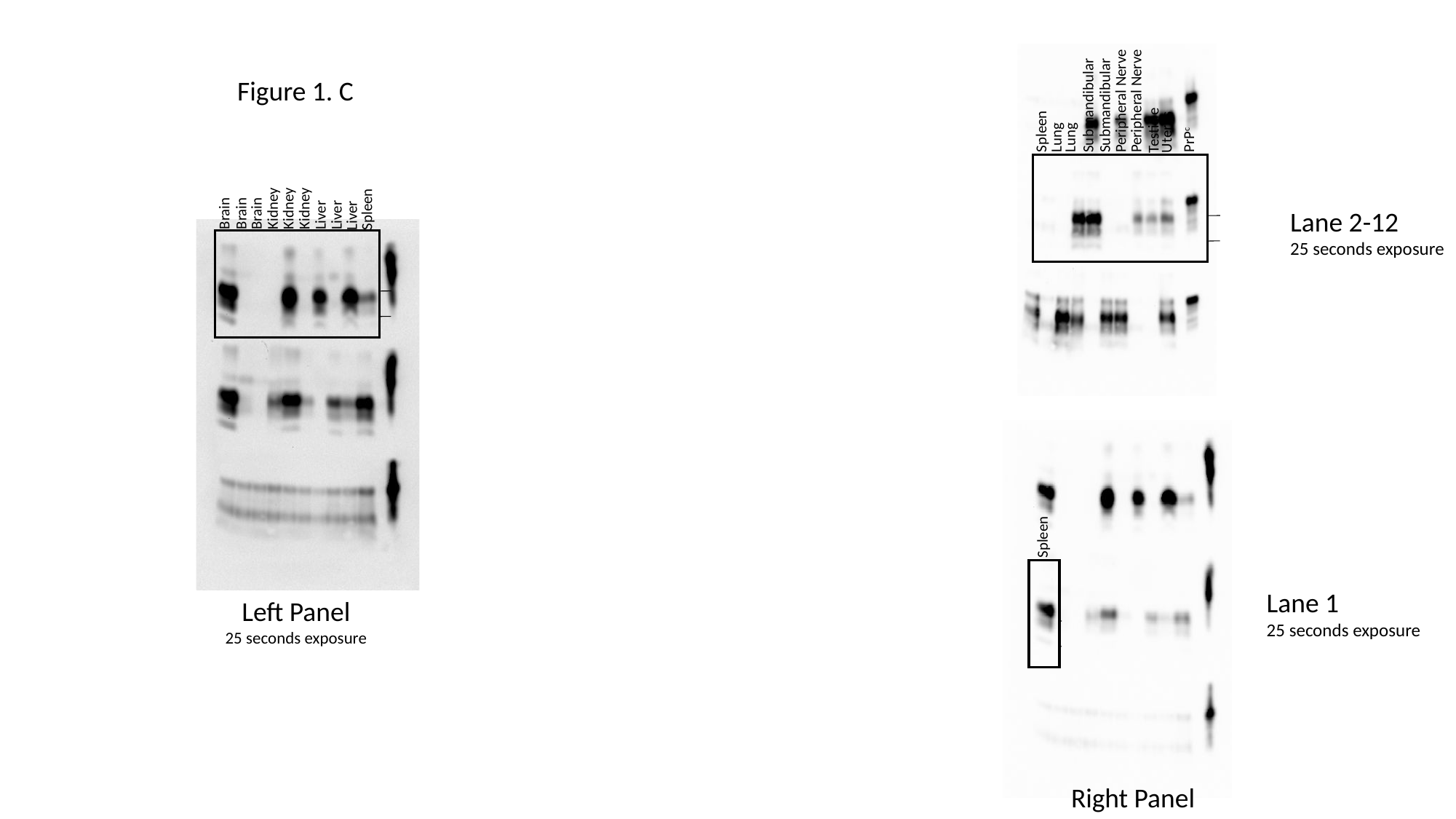

Peripheral Nerve
Figure 1. C
Peripheral Nerve
Submandibular
Submandibular
Spleen
Lung
PrPc
Lung
Testicle
Uterus
Brain
Brain
Brain
Kidney
Kidney
Kidney
Liver
Liver
Liver
Spleen
Lane 2-12
25 seconds exposure
Spleen
Lane 1
25 seconds exposure
Left Panel
25 seconds exposure
Right Panel

## Slide 8
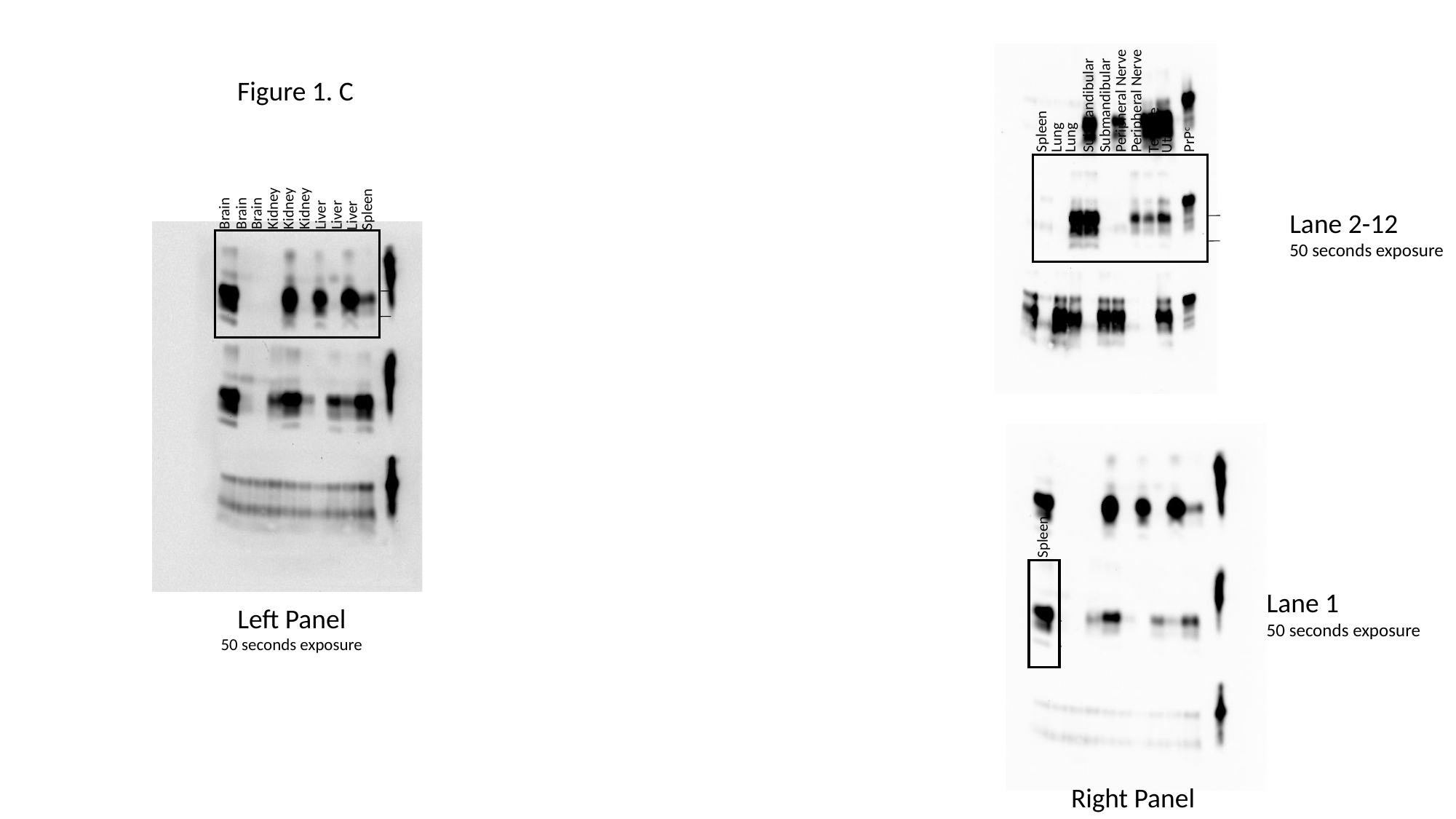

Peripheral Nerve
Figure 1. C
Peripheral Nerve
Submandibular
Submandibular
Spleen
Lung
PrPc
Lung
Testicle
Uterus
Brain
Brain
Brain
Kidney
Kidney
Kidney
Liver
Liver
Liver
Spleen
Lane 2-12
50 seconds exposure
Spleen
Lane 1
50 seconds exposure
Left Panel
50 seconds exposure
Right Panel

## Slide 9
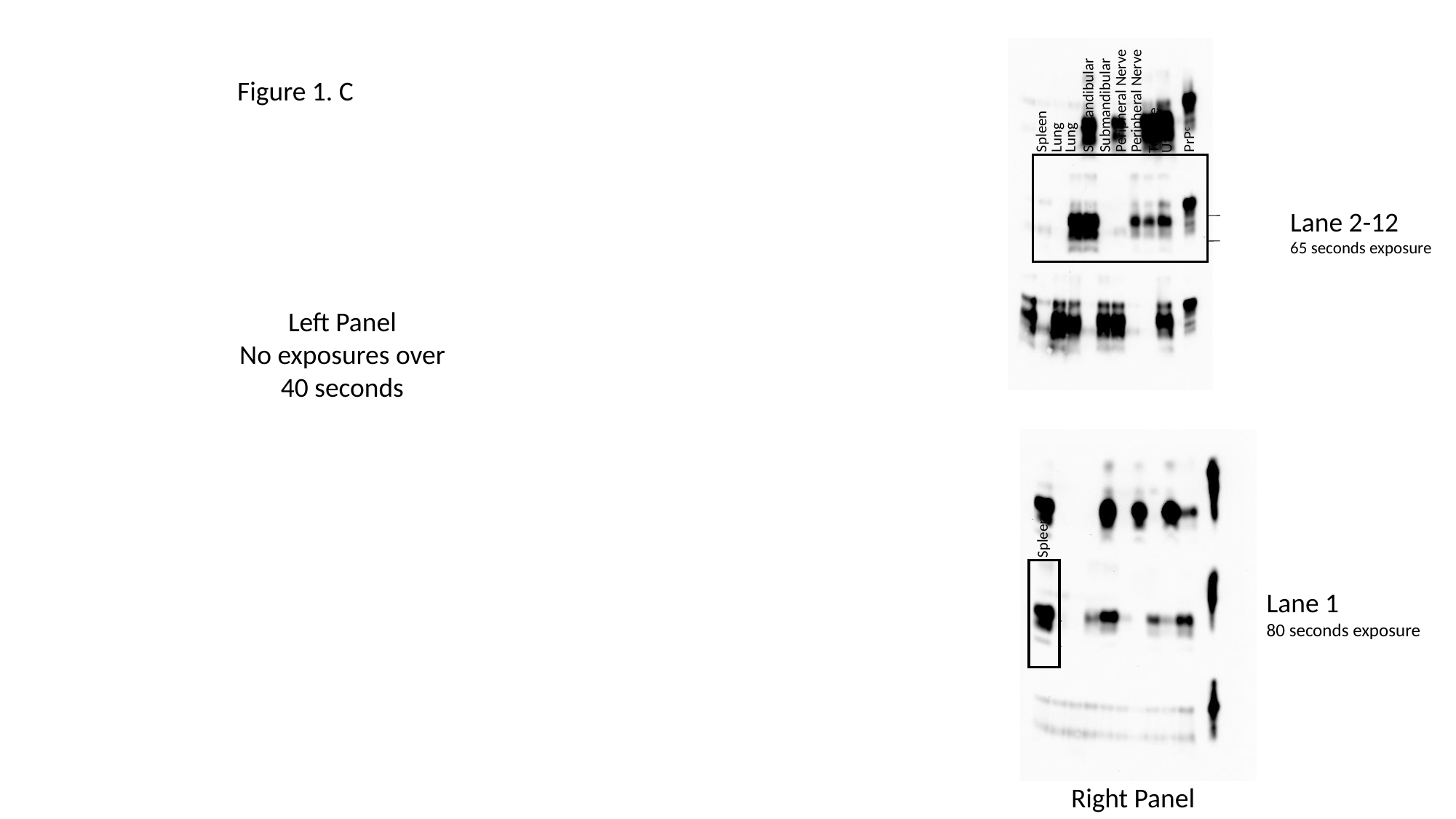

Peripheral Nerve
Figure 1. C
Peripheral Nerve
Submandibular
Submandibular
Spleen
Lung
PrPc
Lung
Testicle
Uterus
Lane 2-12
65 seconds exposure
Left Panel
No exposures over 40 seconds
Spleen
Lane 1
80 seconds exposure
Right Panel

## Slide 10
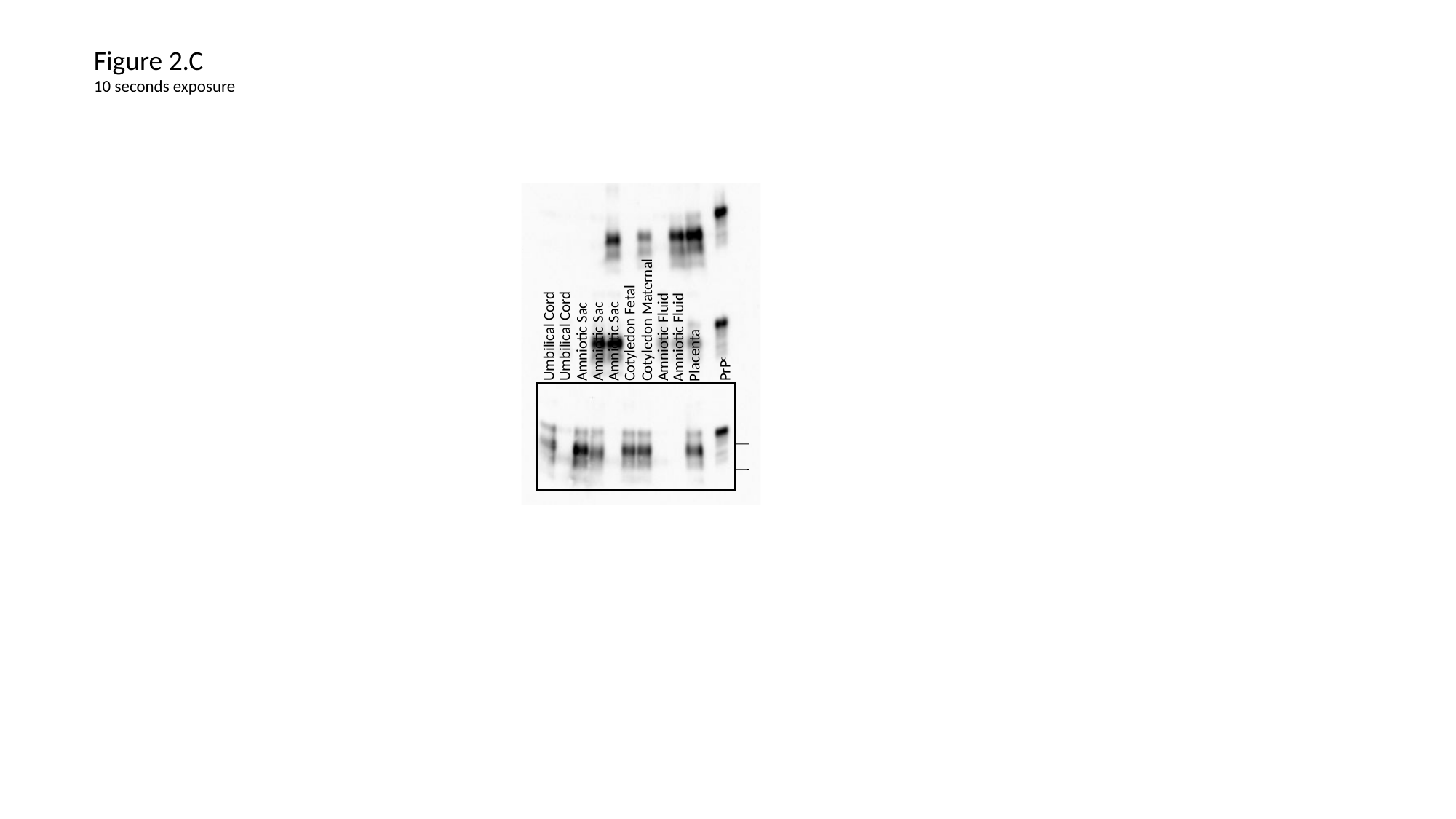

Figure 2.C
10 seconds exposure
Cotyledon Maternal
Amniotic Fluid
Amniotic Fluid
Umbilical Cord
Amniotic Sac
Placenta
Cotyledon Fetal
Amniotic Sac
Umbilical Cord
Amniotic Sac
PrPc

## Slide 11
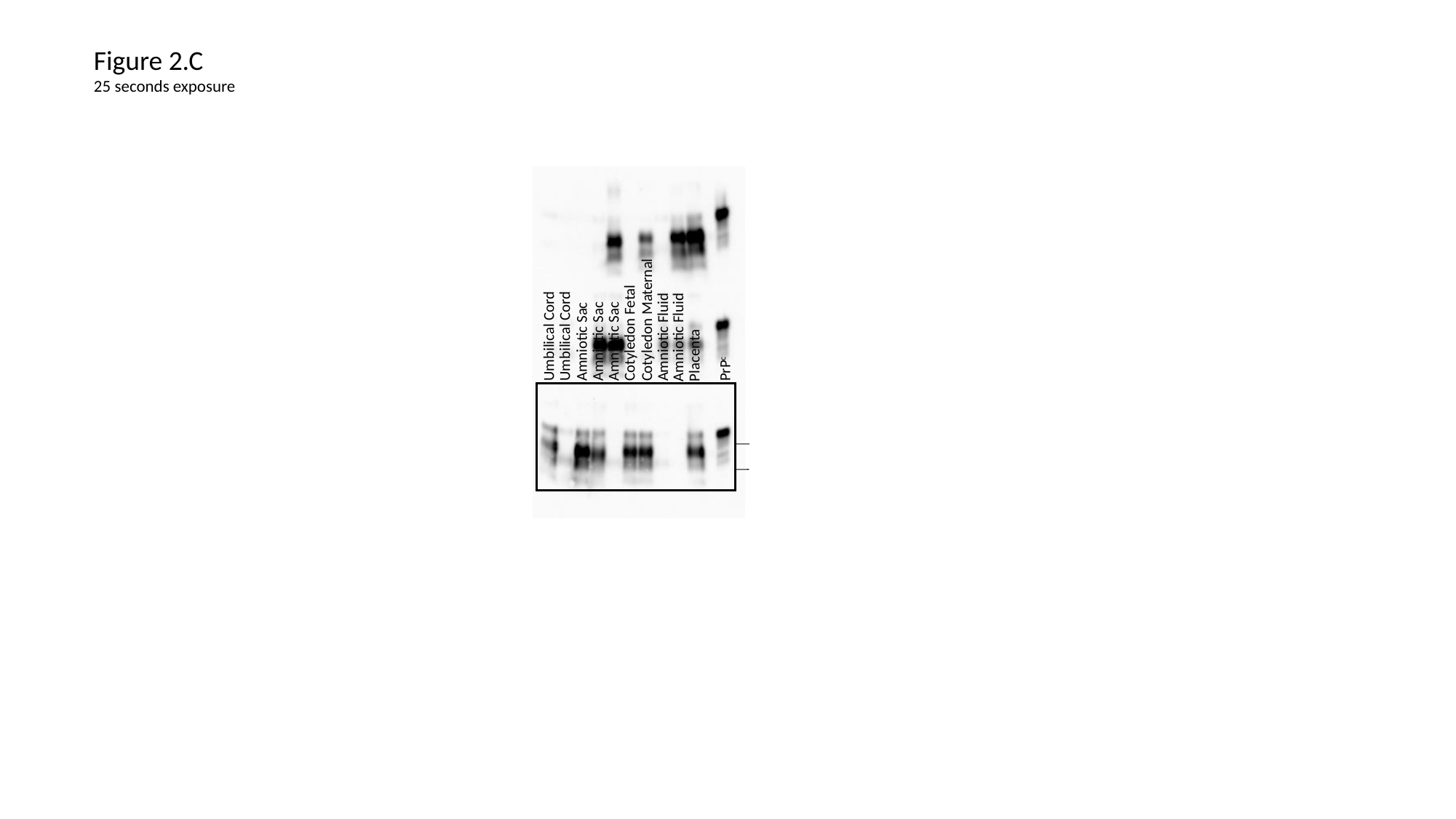

Figure 2.C
25 seconds exposure
Cotyledon Maternal
Amniotic Fluid
Amniotic Fluid
Umbilical Cord
Amniotic Sac
Placenta
Cotyledon Fetal
Amniotic Sac
Umbilical Cord
Amniotic Sac
PrPc

## Slide 12
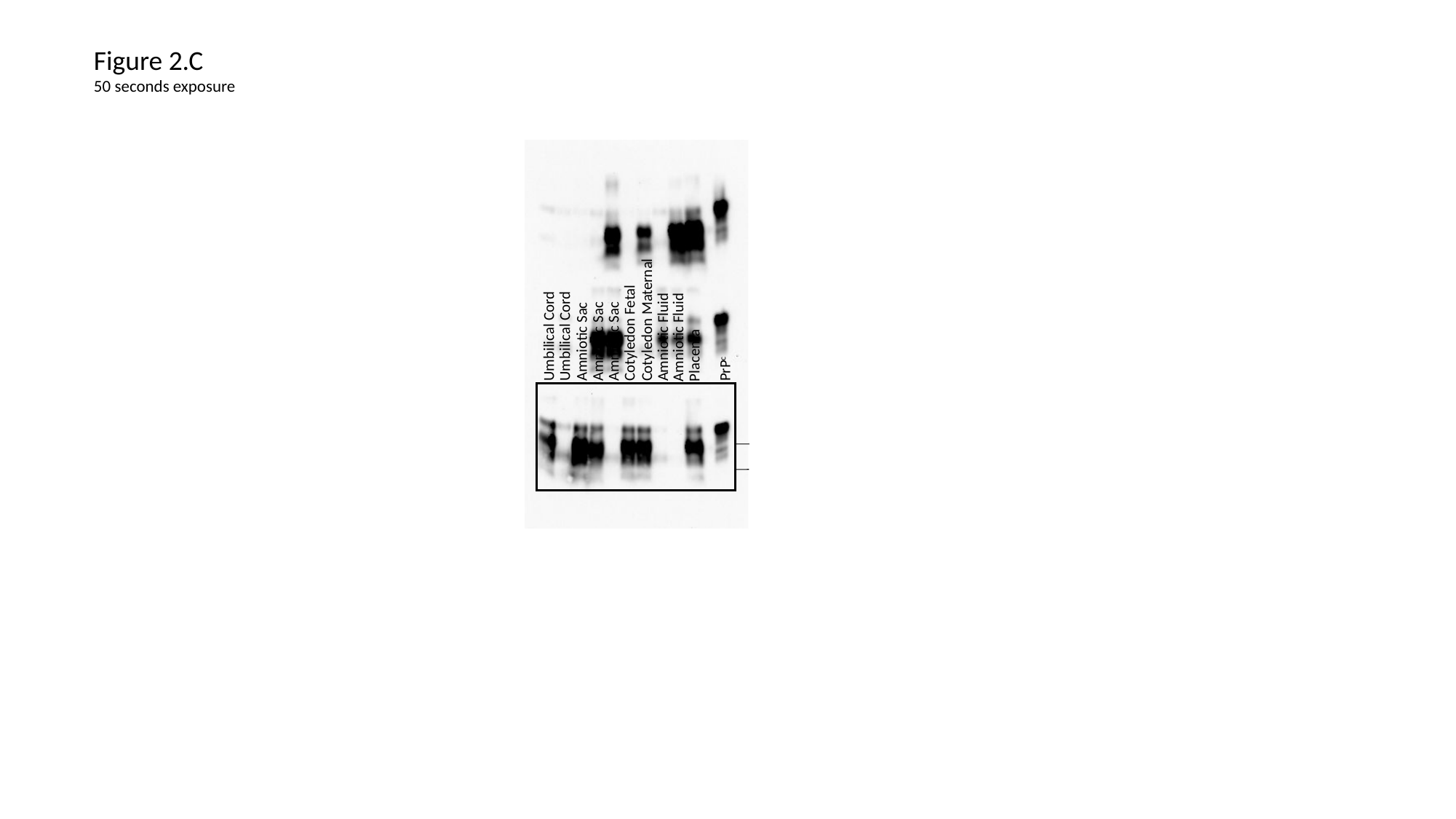

Figure 2.C
50 seconds exposure
Cotyledon Maternal
Amniotic Fluid
Amniotic Fluid
Umbilical Cord
Amniotic Sac
Placenta
Cotyledon Fetal
Amniotic Sac
Umbilical Cord
Amniotic Sac
PrPc

## Slide 13
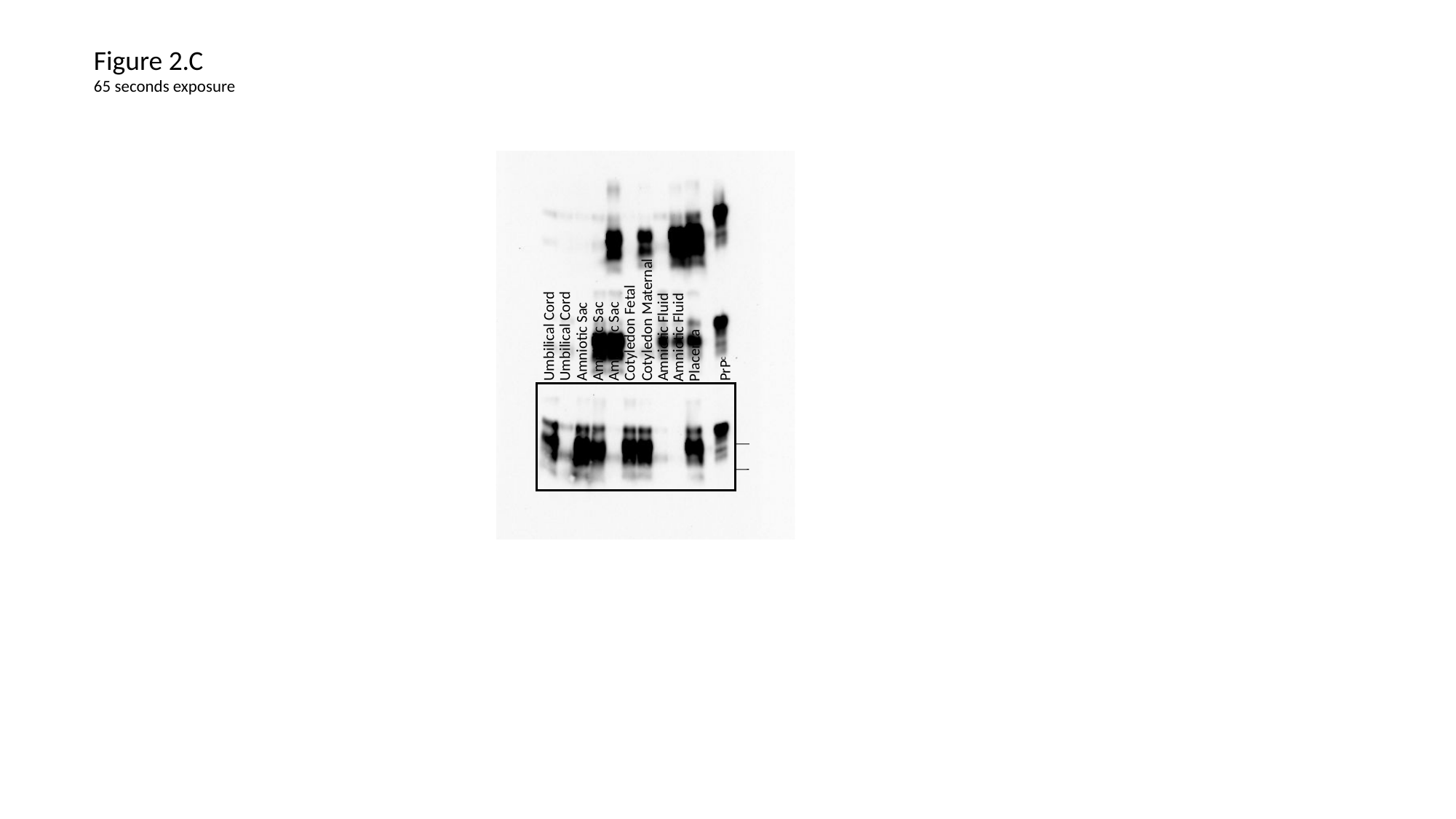

Figure 2.C
65 seconds exposure
Cotyledon Maternal
Amniotic Fluid
Amniotic Fluid
Umbilical Cord
Amniotic Sac
Placenta
Cotyledon Fetal
Amniotic Sac
Umbilical Cord
Amniotic Sac
PrPc
